# Relationship Between Patient Characteristics and Number of Procedures as well as Length of Stay for Patients Surviving Severe Burn Injuries: Analysis of the American Burn Association National Burn Repository

**DOI:** 10.1093/jbcr/iraa040

**Published:** 2020-03-28

**Authors:** Eliza Kruger, Stacey Kowal, S Pinar Bilir, Eileen Han, Kevin Foster

**Affiliations:** 1 IQVIA, Falls Church, Virginia; 2 Arizona Burn Center, Phoenix, Arizona

## Abstract

This study establishes important, national benchmarks for burn centers to assess length of stay (LOS) and number of procedures across patient profiles. We examined the relationship between patient characteristics such as age and total body surface area (TBSA) burned and number of procedures and LOS in the United States, using the American Burn Association National Burn Repository (NBR) database version 8.0 (2002–2011). Among 21,175 surviving burn patients (TBSA > 10–60%), mean age was 33 years, and mean injury size was 19.9% TBSA. Outcomes included the number of debridement, excision, autograft procedures, and LOS. Independent variables considered were: age (linear, squared, and cubed to account for nonlinearity), TBSA, TBSAs of partial-thickness and mixed/full-thickness burns, sex, hospital-acquired infection, other infection, inhalation injury, and diabetes status. Regression methods included a mixed-effects model for LOS and ordinary least squares for number of procedures. A backward stepwise procedure (*P* <0.2) was used to select variables. Number of excision and autografting procedures increased with TBSA; however, this relationship did not hold for debridement. After adjusting for sex, age, and comorbidities, predicted LOS for adults (18+) was 12.1, 21.7, 32.2, 43.7, and 56.1 days for 10, 20, 30, 40, and 50% TBSA, respectively. Similarly, predicted LOS for pediatrics (age < 18) was 8.1, 18.8, 33.2, 47.6, and 56.1 days for the same TBSA groups, respectively. While average estimates for adults (1.12 days) and pediatrics (1.01) are close to the one day/TBSA rule-of-thumb, consideration of other important patient and burn features in the NBR can better refine predictions for LOS.

Approximately 1% of nonfatal injuries among U.S. civilians are burn injuries.^1^ According to recently published estimates, nearly 500,000 burn victims require medical care annually, 40,000 of whom are also hospitalized for burn treatment.^2^ Dramatic improvements have been made in burn care practices over time, resulting in improved clinical outcomes. During the 1960s, burn-related mortality was common for patients with burns of 20% or more of total body surface area (TBSA) given either the initial injury or downstream infections and complications.^3^ Today, the number of burn-related deaths has declined by more than 50% and patients with burns covering up to 90% of their bodies can survive with appropriate management strategies.^3^ While these improvements highlight the benefits of innovation in burn care, there remain opportunities to improve healing and clinical outcomes, thereby reducing patient length of stay (LOS) and the economic burden of burn injuries.^4, 5^ Increased transparency on resource use and the relationship between patient and burn characteristics is a fundamental step in providing a benchmark of real-world care practices. For example, early excision and autografting to achieve definitive closure are recognized cornerstones of modern burn therapy.^6^ Still, there is wide variation in practice, including assessment of depth, timing of eschar removal by wound debridement/excision, extent of excision performed and the products and procedures that are used to achieve definitive closure. Identifying characteristics that drive significant variation in the number of these procedures as well as resulting patient LOS would help care providers understand how their practice compares to overall practices treating a similar patient population.

Although studies have sought to describe treatment trends and predictive relationships in U.S. burn care, robust data detailing the predictive relationship between individual patient characteristics and burn center practice patterns on patient LOS is limited. For example, one study assessed the relationship between burn patient characteristics and operating room visits, number of operations, mechanical ventilation use, and intensive care unit (ICU) days.^7^ In this analysis, the authors included all patients regardless of survival status and injured TBSA and did not consider specific types of procedures. A systematic literature review of publications predicting LOS in thermal burns noted that age and percent TBSA of burn were the strongest predictors of LOS, with percent mixed depth/full-thickness burns, sex, inhalation injury, number of procedures, and depth of burn as additional significant variables.^8^ However, many studies cited in the review focused on smaller TBSA ranges, typically less than 20%.^6, 9–14^ In addition, one publication did not differentiate between surviving patients and nonsurviving patients,^7^ which may confound conclusions.

To the authors’ knowledge, no published research has examined the factors that predict the number of specific types of inpatient procedures and LOS in surviving burn patients in U.S. acute care using real-world data. Therefore, the aim of this study was to examine the relationship between patient characteristics such as age or TBSA of burn on LOS as well as numbers of procedures (debridement, excision, and definitive closure with autografting), using national aggregate data from the American Burn Association (ABA) National Burn Repository (NBR).^15^ Specifically, this article seeks to analyze and develop national aggregate information on procedures used and resulting LOS outcomes by patient and burn type. Leveraging this information, we also seek to develop predictive equations that can be leveraged by the burn community to benchmark individual burn center trends and to inform decisions on the value of new interventions. Furthermore, such information could be leveraged by innovators, key payers, and provider stakeholders to conduct economic evaluations of new burn care interventions.

## METHODS

### Data and Variables

The NBR is a voluntary registry sponsored by the ABA and includes ten years of cumulative data from burn centers, thus representing the largest resource on epidemiology of burn injuries for patients admitted to burn centers in North America.^16^ This study used the NBR version 8.0 (2002–2011) as it was the most up to date version available for analysis.^15^ To avoid confounding factors associated with both mortality and resource use, and to better support hospital financial planning and comparison between specialist centers, we focused on acute care patients with burns covering 10 to 60% TBSA.^17^ Patients with TBSA 61%+ were removed from regression analyses to reduce a tail effect where outlier patients with high TBSA would skew results, as they commonly have exceptionally long LOS and intensive resource use.^7, 17^ We only included surviving patients to reduce confounding in our predictive analysis, as many of the same predictors of mortality (eg, TBSA and inhalation injury) overlap with important predictors of resource use such as LOS.^18^

Key outcomes of interest were the number of procedures identified via ICD-9 code, specifically including nonexcisional debridement (debridement, 86.28), excisional debridement (excision, 86.22), and autograft (86.60, 86.61, 86.62, 86.63, and 86.69), and NBR-reported LOS. As autograft procedures were identified by multiple codes, we assumed that multiple unique codes were applied in the same surgical intervention (ie, assumed to represent a unique and single operating room visit). Therefore, the maximum count of any individual ICD-9 code avoids double-counting and thus avoids overestimation of the number of autografting procedures. Please note that while number of operating room procedures is variable in the NBR, less than one-quarter (21%) of our analysis sample has this variable populated. Therefore, within this analysis, we assumed that presence of the aforementioned ICD-9 codes can be interpreted as a surgical intervention, which we referred to as a procedure throughout this article.

Independent variables were informed by a review of the published burn literature,^7, 8^ interviews with burn surgeons and availability of variables in the NBR. These variables included patient characteristics such as age (in years), sex, selected comorbidities (diabetes status, hospital-acquired infection [HAI], other infection, and inhalation injury), burn TBSA, and whether the burn was superficial partial-thickness (SPT, defined as patients expected to heal in less than 14 to 21 days without an autograft procedure). To account for possible nonlinear relationship between the outcomes and the independent variables, the squared and cubed forms of age and TBSA were also included in the model.

### Analyses

Descriptive statistics were calculated for all variables, stratified by patient age group (pediatrics: age 0–17 years or adult: age 18 years or older). For continuous variables, the mean was reported; for categorical variables, the proportion of patients observed in each category was reported. All analyses were conducted using Stata Version 15.^19^

Dependent variables were number of procedures (per the ICD-9 codes noted above) and LOS. A backward selection stepwise process was used to identify independent variables included in each analysis. Independent variables were removed from the model if the level of significance exceeded 20% (ie, * P* > 0.2). To predict the number of procedures—debridement, excision, and autograft—ordinary least squares (OLS) models were fitted. Only patients with one or more autografting procedures were included in the analyses for number of autografting procedures. A mixed-effects linear model was selected for LOS to adjust for patient case mix and hospital characteristics and to account for the dependence of outcome variables within hospitals. This regression method was considered the most appropriate based on a review of similar regression analyses of LOS of the literature.^20–22^

Descriptive statistics of mean patient and burn characteristics for pediatrics and adults for TBSA 10, 20, 30, and 40% were multiplied by regression coefficients to generate predicted results for average number of procedures and LOS for a range of patient profiles to allow for benchmark comparisons. Output of the regression could be applied to any burn patient via use of coefficients applied to the patient’s unique burn and demographic characteristics (see Table 2). To help put the output of the regression in context, we used regression output and average patient characteristics from the NBR to estimate outcomes for TBSA ranges of 10 to 60%. Please note that results for 60% and over are not reported given that the last included TBSA band was 50 to 59%.

**Table 2.** Regression model coefficients

	Debridement (OLS)			Excision (OLS)			Autograft (OLS)			LOS (mixed effects)		
Coefficients	Beta	SE	*P*	Beta	SE	*P*	Beta	SE	*P*	Beta	SE	*P*
TBSA	−0.081	0.020	<0.001	0.031	0.029	0.276	0.068	0.003	<0.001	0.708	0.161	<0.001
TBSA^2^	0.002	0.001	0.001	0.001	0.001	0.454				0.015	0.006	0.012
TBSA^3^	0.000	0.000	0.003	0.000	0.000	0.662				0.000	0.000	0.087
TBSA PT	0.009	0.002	<0.001	−0.031	0.003	<0.001	−0.023	0.003	<0.001	−0.565	0.016	<0.001
Age				0.008	0.003	0.007	−0.010	0.005	0.030	−0.275	0.039	<0.001
Age^2^	0.000	0.000	0.014	0.000	0.000	0.068	0.000	0.000	0.014	0.009	0.001	<0.001
Age^3^	0.000	0.000	0.005							0.000	0.000	<0.001
Female	−0.065	0.032	0.042				0.130	0.068	0.056	1.811	0.262	<0.001
HAI	−0.110	0.085	0.196	0.608	0.125	<0.001				11.269	0.757	<0.001
Other infection							−0.282	0.140	0.045	4.110	0.683	<0.001
Inhalation injury							0.210	0.091	0.021	7.563	0.399	<0.001
Diabetes	0.331	0.079	<0.001	0.564	0.116	<0.001	0.430	0.156	0.006	1.880	0.656	0.004
SPT	0.659	0.030	<0.001	−1.676	0.045	<0.001				−9.169	0.267	<0.001
Constant	1.053	0.156	<0.001	1.872	0.230	<0.001	1.886	0.101	<0.001	11.108	1.465	<0.001
	*N* = 21,175			*N* = 21,175			*N* = 12,333*			*N* = 21,175		
	*R* ^2^ = 0.03			*R* ^2^ = 0.13			*R* ^2^ = 0.04			*R* ^2^ = 0.43		

*OLS*, ordinary least squares, *LOS*, length of stay; *SPT*, superficial partial-thickness; *HAI*, hospital-acquired infection.

*Sample size is reduced for autografting given requirement that patients in the sample for this concept received an autograft.

## RESULTS

From NBR 2002 to 2011 data, a sample of 21,175 surviving patients, with nonmissing data for dependent and independent variables, was identified. Table 1 provides the description of the sample (descriptive statistics by TBSA are available in the Supplementary Appendix). Among the final sample, the average age of surviving burn patients was 32.8 years, approximately one-third were female, and the average TSBA was 19.9%. Viewing the descriptive data in aggregate without any regression analyses or adjustments, the mean LOS per TBSA percent was 0.84 for pediatrics and 1.09 for adults. Compared with adult patients, pediatric patients had a higher proportion of females (35% vs 24%), higher proportion of mixed depth/full-thickness burn (37% vs 33%), and fewer excision and autografting procedures (excision, 1.7 vs 2.0; autograft, 1.7 vs 1.8;). Pediatric patients also had a higher proportion of patients with SPT (47% vs 40%). This trend is not unexpected, as epidemiology reports of burn injury indicate a high incidence of scalds in pediatrics.^17^ As such, this higher proportion of SPT burns in pediatrics could be driven by a greater proportion of scalds, which is consistent with the noted lower rate of inhalation injury (6% vs 12%).

**Table 1.** Demographic characteristics of surviving burn patients

	Pediatrics	Adult	
	(0–17 years)	(18+ years)	All patients
Number of patients (*n*)	5957	15,218	21,175
Mean age at time of burn injury (years)	6.3	43.2	32.8
Sex			
Female (%)	35%	24%	27%
Male (%)	65%	76%	73%
Comorbidities			
Inhalation injury	6%	12%	10%
HAI	2%	3%	3%
Other infection	2%	4%	4%
Diabetes	0%	4%	3%
Characteristics of burn			
Total TBSA (%)	19.8%	19.9%	19.9%
Partial thickness TBSA (%)	12.4%	13.3%	13.1%
Full-thickness TBSA (%)	7.4%	6.6%	6.8%
Proportion patients SPT (%)	47%	40%	42%
Number of procedures			
Debridement	0.7	0.7	0.7
Excision	1.7	2.0	1.9
Autograft	1.7	1.8	1.8
LOS (days)			
Average	17.4	22.0	20.7
Per percent TBSA	0.84	1.09	1.02

*HAI*, hospital acquired infection; *LOS*, length of stay; *SPT*, superficial partial-thickness; *TBSA*, total body surface area.

Descriptive statistics for the final sample are provided above. LOS reported above is mean values for the sample and is not adjusted for patient characteristics.

Table 2 shows the estimated coefficients for each parameter in the regression model for LOS. All independent variables were retained in the model. The Bryk and Raudenbush^23^*r*^2^ level 1 of the predicted model was 0.43, suggesting that the model had an adequate fit given underlying data. Examining the impact of TBSA alone, all else being equal among other independent variables, TBSA contributes to some but not all of LOS, with each additional percent TBSA leading to approximately 0.723 more days of LOS. Given the coefficients observed across other independent variables, other factors also played a key role in predicting LOS for a given patient. For example, presence of HAI was almost as influential as TBSA for debridement and could be more important than TBSA for smaller burns when predicting number of excisions or LOS. Furthermore, while age was not a strong predictor of number of procedures, it had a notable impact on LOS. While the exact impact of each independent variable such as TBSA, age, sex, HAI, and diabetes, is dependent on each patient’s unique characteristics, the difference in magnitudes for the coefficients for each independent variable shows important variation across key procedures and LOS.

Table 3 presents the model predictions for each outcome. As noted above, mean patient and burn characteristics across TBSA ranges from the NBR were leveraged to translate the regression findings into benchmark information for comparison. Details on the patient and burn characteristics for each TBSA range are reported in Supplementary Appendix. While TBSA was a significant variable for debridement, overall number of debridement procedures did not increase with TBSA for either adults or pediatrics, as the impact of TBSA was outweighed by other factors. However, the number of excision procedures did increase with TBSA. When a definitive closure (ie, autografting) was required, the predicted number of autograft procedures was similar for adults and pediatrics, with the number of autograft procedures increasing as TBSA burned increased.

**Table 3.** Predicted number of debridement, excision, autograft procedures, and LOS by age group and TBSA

	Debridement Procedures (*n*)	Excision Procedures (*n*)	Autograft Procedures (*n*)	LOS (days)
**Adults (18+)**				
TBSA (%) Burned				
10%	1.0	1.3	2.3	12.1
20%	0.7	1.9	2.8	21.7
30%	0.6	2.5	3.4	32.2
40%	0.7	3.1	4.0	43.7
50%	0.4	3.9	4.8	57.5
**Pediatrics (0–17)**				
TBSA (%) Burned				
10%	1.0	0.9	2.4	8.1
20%	0.6	1.7	2.9	18.8
30%	0.5	2.5	3.6	33.2
40%	0.5	3.4	4.3	47.6
50%	0.6	3.8	4.6	56.1

Estimates above represent averages for the population with each burn depth, with patient characteristics informed by the final analysis sample from the NBR. Please see Supplementary Appendix for more detail on average patient characteristics by age group and TBSA range.

**Figure 1.**
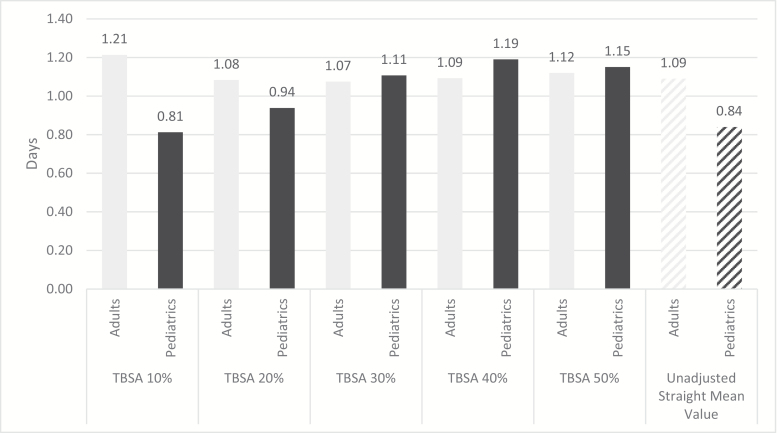
Average predicted LOS days per percent TBSA for adults and pediatrics for surviving patients. Columns represent number of inpatient days per percent TBSA burned. Adult patients (18 and older) represented in light gray and pediatrics (17 and under) in dark gray. X-axis labels denote group of patients based on TBSA burned. Adjusted estimates for LOS per percent TBSA based on regression analysis are also shown alongside average, unadjusted estimates for patient characteristics in this sample was leveraged to generate average LOS estimate. Columns with gradient fill (for adults, pediatrics) show the straight mean. Please note that these estimates were derived without rounding.

After adjusting for sex, age, and comorbidities, predicted LOS for adults (age 18+) was 12.1, 21.7, 32.2, 43.7, and 57.5 days for 10, 20, 30, 40, and 50% TBSA, respectively. For pediatrics (age < 18), the predicted LOS was 8.1, 18.8, 33.2, 47.6, and 56.1 days for 10, 20, 30, 40, and 50% TBSA, respectively. When considering the impact of all independent variables, the average LOS per percent TBSA is estimated at approximately 1.12 and 1.01 days for adults and pediatrics. For pediatrics, the average LOS days per percent TBSA increased with TBSA, from 0.81, 0.94, 1.11, and 1.19 days for 10, 20, 30, and 40%. For adults, LOS days per percent TBSA increased by 1.21, 1.08, 1.07, and 1.09 days for TBSA 10, 20, 30, and 40%, respectively. Trends for 50% TBSA showed a continued increase in days per percent TBSA for adults (1.12) but a slight decreasing trend for pediatrics (1.15). However, this information should be interpreted with caution given expected confounding in this high TBSA category. Although the observed LOS in pediatrics is in general lower than adults, the overall trend of increasing LOS with increasing percent TBSA of burn were similar. As the percent TBSA burned increases, the relative impact on LOS also increases to become one of the dominant factors influencing LOS outcomes. Figure 1 further illustrates these findings and compares adjusted estimates for LOS per percent TBSA to unadjusted mean values from the NBR.

**Figure 2.**
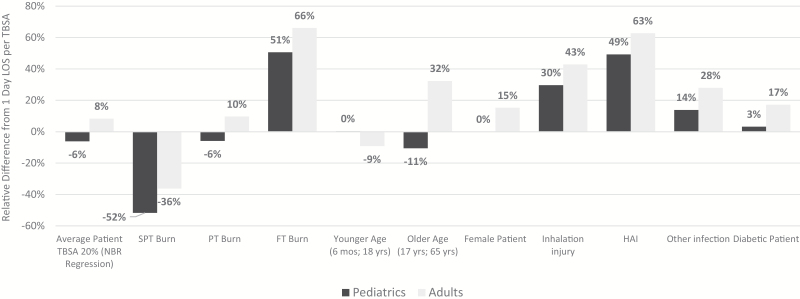
Relative difference from 1 day LOS per TBSA across varying patient characteristics (scenario analysis: adult or pediatric patient with 20% TBSA burned). Columns represent relative difference (percent change) from common rule of thumb baseline of 1 day per TBSA (0% Y-axis represents 1 day per TBSA rule of thumb). Adults patients (18 and older) represented in light gray and pediatrics (17 and under) in dark gray. X-axis labels denote scenario tested to show variation across patient characteristics, including: average from the NBR, patients with an SPT burn, PT or FT burn, Patients at younger and older ages within population range, sex, and presence of key comorbidities (ie, inhalation injury, HAI, other infection, and diabetes). Numbers above 0% represent increase beyond 1 day per TBSA and negative figures represent lower than 1 day per TBSA. For example, increase of 51 and 66% for pediatrics and adults, respectively, indicate that LOS was 51 and 61% greater than 1 day per TBSA for patients with full thickness burns.

Although the 1 day per percent TBSA rule of thumb may somewhat approximate LOS, the key benefit of generating a predictive equation from regression analysis is the ability to capture the impact of many influential characteristics that work in a multifactorial fashion to predict LOS outcomes. The ability of TBSA alone to accurately predict LOS is indeed variable based on underlying patient characteristics. For example, when evaluating how LOS may present for an individual patient, the range of difference from the 1 day per TBSA rule is more notable. Figure 2 shows the relative difference (expressed as percent change) from the 1 day per TBSA common clinical approximation across potential patients with 20% TBSA. Moving away from a weighted average of the NBR population characteristics, we can see how LOS changes based on sex (male, female), actual age (0.5 to 17 years for pediatrics; 18 to 65 years for adults), burn depth, and presence of comorbidities. Considering the estimated LOS with the 1 day per TBSA approximation is 20 days for a patient with 20% TBSA burned, differences in individual patient characteristics, such as full-thickness depth of injury, can drive up to a 66% shift in LOS, or up to a change in LOS of 13.2 days (20 days for rule of thumb compared to 33.2 days).

## DISCUSSION

This study represents the first analysis that develops real-world evidence-based predictive equations to explore the relationship between patient characteristics and LOS as well as three specific procedures among surviving burn patients with TBSA 10% or more in the United States. When controlling for typical average patient characteristics as captured in the NBR, we find LOS per percent TBSA is estimated at approximately 1.12 days per percent TBSA for adults and 1.01 for pediatrics, with average LOS per percent TBSA increasing with TBSA. While TBSA was found to be a significant predictor of excision and autograft procedures as well as LOS, it is not the only factor that affects these outcomes. Patient age, sex, comorbidities, and burn characteristics beyond TBSA may be as important. Notably, large positive coefficients for HAI, infections, and inhalation injury, as well as a large negative coefficient for SPT burns can influence predicted LOS. Furthermore, the coefficient for partial thickness is almost as large as the coefficient for TBSA,, indicating that the depth of burn is an important feature when predicting LOS. Considering additional burn and patient characteristics, the existing 1 day per TBSA rule of thumb could differ from expected LOS by 60% or more, as illustrated by the 20% TBSA example described above.^13^

Our findings are consistent with summary descriptive statistics provided in the 2017 ABA NBR report.^17^ Specifically, summary statistics of all NBR patients (regardless of burn size) by age found unadjusted number of hospital days per percent TBSA exceeded 1 for all surviving patients (all TBSAs), with a low of 1.66 days per percent TBSA for infants aged 12 to 23 months ranging up to 3.94 days per percent TBSA for adults aged over 80.^17^ The higher average LOS per percent TBSA from the NBR sample is likely due to the floor effect of including smaller TBSA burns (ie, inpatient days are greater than 1, even for small burns). These findings suggest that a more nuanced approach to accurately estimate LOS is needed, and that considering patient and burn characteristics (in particular, age and depth of burn) is needed in addition to TBSA.

While TBSA is a significant predictor for debridement, there is no discernable increase in the number of debridement procedures with increasing TBSA. It is expected that unknown factors or differences in clinical practice may play a larger role in determining the number of nonexcisional debridement procedures required. For example, eschar removal via excision may have been preferred for patients subsequently receiving an autograft, while nonexcisional debridement may have been preferred for burns not needing autografting, diluting the impact of TBSA on overall debridement trends. Furthermore, noting the high number of autografting procedures relative to the number of excision procedures, this trend may be impacted by either coding practices (as this analysis had to rely in ICD-9 codes given the nature of the dataset) or the potential for excision to be done in tandem with grafting in a single procedure, captured under autografting and not excision codes. Finally, please note that while the debridement and excision may be used interchangeably, this analysis assumes that the definitions align with the ICD9 codes available in the NBR. This is a limitation given that we can only report on trends for these procedures based on coding, which may be subject in inaccuracy. Therefore, a future area of research could leverage survey data to better understand the relationship between use of these procedures in practice and coding approaches to better place the results of our findings in context.

In a previous regression analysis that included both surviving and deceased burn patients in its sample,^7^ the authors found that size and severity of burn, inhalation injury, and age were significant predictors of resource utilization, such as the number of operative procedures (ie, sum of ICD-9 procedure codes), total operating room visits (ie, multiple procedures may be performed in the same operating room visit), LOS in ICU, and length of time on a ventilator. In our analysis of only surviving burn patients, the above variables were also significant predictors of excision, debridement, and autograft procedures and LOS. In addition, depth of burn and sex were significant predictors in our model, which is consistent with previous literature.^7, 8^ Overall, our research builds on the body of evidence that concludes consideration of burn and patient characteristics (beyond just TBSA) supports better predictions of resource utilization, regardless of the type of patients ultimately included in the analysis (eg, severe burn patients and/or deceased patients). This research aimed to establish initial predictive equations by looking at surviving burn patients and limiting to smaller burns. Indeed, while we report burns of more than 50% TBSA, it should be noted that the influence of confounders in this population may have a more substantial impact on outcomes than other variables. Therefore, an area of future research could be to repeat this analysis across different samples of patients (such as survivors, those who died late in therapy, large burns) to more formally assess trends in these populations as well as the shifting importance of predictive variables. Additionally, interpretation of this research, as well as application of this research in practice, should remain aware of the challenges of treating many complex patients in burn care who may have other comorbid conditions beyond those captured in the NBR that impact outcomes.

This analysis focused on understanding resource utilization of a different patient cohort than has been examined previously. Specifically, our patient sample includes large burns, surviving patients, and focused regression analysis on a TBSA range of 10 to 60% to reduce the biasing effect of outliers. Additionally, these analyses sought to consider specific types of procedures, adding granularity on key intervention and resource use detail during an inpatient stay. Finally, this analysis provides a more nuanced estimate of LOS days per percent TBSA for surviving burn patients, highlighting the differences between average LOS per percent TBSA between pediatrics and adults and when adjusting for typical patient characteristics.

Summary information on national aggregate trends for procedures and LOS may be useful for burn surgeons and centers seeking to benchmark resource utilization and center outcomes. This analysis may also facilitate assessment of new interventions/medical countermeasures (MCMs) by setting a baseline of resource use associated with current standard of care in this patient cohort. Accordingly, any new intervention that aims to lessen resource utilization may be evaluated against this threshold. For example, international real-world studies have examined the impact of type of skin replacement surgery on LOS and surgery time outcomes to assess real-world effectiveness.^24, 25^ Furthermore, the predictive equations derived in this study were leveraged in an economic model, the Burn-MCM Effectiveness Assessment Cost Outcomes Nexus (BEACON) model, to predict costs, outcomes and the value of new innovations for burn care patients in the United States.^26, 27^

This study has several limitations that should be noted when interpreting and using the results. For models predicting the number of procedures, low *r*^2^ values indicate that the model specifications describe only a small proportion of the variation in the numbers of procedures. This suggests that other unknown factors, such as variation in hospital practices that affect outcomes but are not captured in the dataset, may have an important influence. For example, some burn surgeons may take a more “wait-and-see” approach to burn wounds of indeterminate depth, whereas others may excise and autograft the burn wound at the earliest possible opportunity.^28^ In the former case, the surgeon may nonsurgically debride the wound to assess whether the wound may be treated conservatively and may therefore have more debridement procedures compared to the latter approach.^29^ Furthermore, related to differences in practices on timing of intervention, the true depth of burn is subjective and potentially unreliable.^30^ Currently, the NBR does not code depth of burn at multiple time points, which limits the ability to capture potentially important changes in burn depth diagnosis as surgeons learn more over time. Therefore, while this study uses the best available data from the NBR, this limitation should be noted when interpreting findings. Another key finding of this research is that burn care practices have additional relatively uncontrolled influencing factors important in driving these outcomes than patient characteristics. However, at present, the NBR does not include information attributed to region or individual burn centers to allow for formal consideration of these factors in regression analysis. Therefore, while the presented results are a foundational step to establish a baseline understanding of outcomes across key patient characteristics, an important area of future research will be to more formally evaluate how individual burn center practices may improve patient resource utilization-related outcomes.

In addition, the mixed-effects model for prediction of LOS exhibited greater variability in outcomes for increasing TBSA (ie, heteroscedasticity). Despite an attempt to mitigate this by transformation of LOS to the logarithmic domain, the issue largely remained and, further, predictive bias was introduced during back transformation.^31^ This mixed-effect specification reflects the greater variability in outcomes observed in the treatment of larger burns, wherein compounding clinical issues can sometimes have substantial impacts on LOS.

Finally, it is important to note the data analysis utilized ABA NBR Version 8.0, which includes burn patients from 2002 to 2011. Trends may have accelerated or changed since this time, which is not discernable due to the lag in data availability. Version 8.0 was the most recent dataset available at the time of analysis, and thus this study reflects the most up-to-date analysis possible. Given the recent release of the 2019 update to the NBR research dataset, future work could repeat this regression analysis to provide updated predictive equations and to compare trends in influential independent variables over time. Furthermore, it should be noted that while this study uses one of the most robust and rich datasets for burns available in the United States, the NBR is not exhaustive in its inclusion of important factors known to impact patient outcomes, including LOS. For example, recent retrospective analysis of electronic medical records found that other important variables, including but not limited to socioeconomic status and key comorbid conditions beyond burns (clotting disorders, anemia, and admission serum ethanol level) are important variables associated with LOS not captured in the NBR.^32^ Similar to the conclusions of the Smith et al study, we also recommend that variables in the NBR be expanded to capture more important concepts that help to better understand predictive factors for patient outcomes.

## CONCLUSIONS

This analysis uses regression of national aggregate data from NBR to examine the predictive relationship between patient and burn characteristics and number of procedures (debridement, excision, and autograft) as well as LOS for surviving, severely burned patients. A key finding of this analysis is that the general rule of assuming one day of inpatient day per percent TBSA likely underestimates LOS for adults and pediatrics with large burns and overestimates for pediatrics with TBSA less than 20%, given a lack of consideration of other important factors such as HAI, exact patient age, depth of burn, sex, and inhalation injury.

These estimates also provide a benchmark against which burn centers can compare the number of procedures and LOS for various patient profiles (eg, per age, TBSA). By identifying patients with characteristics that lead to excess resource use, burn centers can examine more closely why these patients need more care, creating opportunities for more tailored care practices based on patient and burn characteristics. Furthermore, these model equations permit burn centers to evaluate their own performance and highlight any potential areas for improving efficiency. These estimates can also indicate whether a given burn center achieves definitive closure with shorter LOS and fewer procedures. These predictive equations also provide second-order information, as comparative value for cost of interventions can be evaluated by feeding the equations into a larger burn economic model.^26, 33^ Finally, it may be feasible to predict costs and resource utilization at a population or regional level, according to patient mix and expected interventions, supporting a higher level understanding of the anticipated impact of potential changes or new interventions in burn care.

## Supplementary Material

iraa040_suppl_Supplementary_Appendix

## Notes

Funding: This work was funded by Biomedical Advanced Research Development Authority, AVITA Medical. This work was supported under the HHS/ASPR/BARDA Contract no. HHSO100201500028C.
