# Supplementary material for: Relationship Between Patient Characteristics and Number of Procedures as well as Length of Stay for Patients Surviving Severe Burn Injuries: Analysis of the American Burn Association National Burn Repository
Source: J Burn Care Res. 2020 Mar 28;41(5):1037–44. doi: 10.1093/jbcr/iraa040 (PMC7510847; doi:10.1093/jbcr/iraa040)
Supplement: iraa040_suppl_Supplementary_Appendix [file iraa040_suppl_supplementary_appendix.docx]

**Supplementary Appendix Table 1:** Demographic characteristics of surviving burn patients by TBSA group

|  | TBSA 10% | | TBSA 20% | | TBSA 30% | | TBSA 40% | | TBSA 50% | |
| --- | --- | --- | --- | --- | --- | --- | --- | --- | --- | --- |
|  | Pediatrics | Adult | Pediatrics | Adult | Pediatrics | Adult | Pediatrics | Adult | Pediatrics | Adult |
|  | (0-17 years) | (18+ years) | (0-17 years) | (18+ years) | (0-17 years) | (18+ years) | (0-17 years) | (18+ years) | (0-17 years) | (18+ years) |
| Mean age at time of burn injury (years) | 5.29 | 43.77 | 6.59 | 45.01 | 6.74 | 43.47 | 7.50 | 38.36 | 8.20 | 39.13 |
| Sex |  |  |  |  |  |  |  |  |  |  |
| Female (%) | 38% | 26% | 32% | 24% | 39% | 25% | 31% | 23% | 33% | 21% |
| Male (%) | 62% | 74% | 68% | 76% | 61% | 75% | 69% | 77% | 67% | 79% |
| Comorbidities (%) |  |  |  |  |  |  |  |  |  |  |
| Inhalation injury | 1% | 5% | 5% | 9% | 17% | 13% | 27% | 26% | 16% | 36% |
| HAI | 0% | 1% | 2% | 3% | 2% | 4% | 2% | 6% | 5% | 14% |
| Other infection | 1% | 2% | 3% | 5% | 5% | 5% | 2% | 5% | 4% | 10% |
| Diabetes | 0% | 6% | 0% | 6% | 0% | 3% | 0% | 4% | 0.5% | 4% |
| Characteristics of burn |  |  |  |  |  |  |  |  |  |  |
| Total TBSA (%) | 10.00% | 10.00% | 20.00% | 20.00% | 30.00% | 30.00% | 40.00% | 40.00% | 50.00% | 50.00% |
| Partial thickness TBSA (%) | 8.38% | 7.60% | 12.85% | 13.82% | 13.68% | 18.59% | 13.76% | 21.85% | 19.86% | 29.08% |
| Full-thickness TBSA (%) | 1.85% | 2.66% | 7.77% | 6.58% | 16.85% | 11.96% | 27.11% | 19.17% | 35.29% | 26.51% |
| Proportion patients SPT (%) | 69% | 56% | 45% | 41% | 29% | 32% | 21% | 34% | 19% | 24% |

**Supplementary Appendix Table 1**: Demographic Characteristics of Surviving Burn Patients by TBSA Group. Descriptive statistics for the final sample are provided above, with detail shown for each TBSA range reported in the results section. HAI - hospital acquired infection; LOS, length of stay; SPT, superficial partial-thickness; TBSA, total body surface area. LOS reported above is mean values for the sample and is not adjusted for patient characteristics.
